# 

**Published:** 1850-09

**Authors:** 


### CONTENTS

OF THE

### MEDICAL EXAMINER.

NEW SERIES—NO. LXIX.—SEPTEMBER, 1850.

### ORIGINAL COMMUNICATIONS.

On Acclimation. An Inaugural Essay presented for the degree of Doctor of Medicine in the University of Pennsylvania. By Philip C. Williams, M. D., of Winchester, Virginia, (Concluded,) - - - - - - - 499

Case of Extra-Uterine Pregnancy. Reported by Carter P. Johnson, M. D., Professor of Anatomy and Physiology in the Medical Department of Hampden Sidney College, Richmond, Va., - 511

Strychnia in Intermittent Fever. By O. H. Edwards, M. D., of Clinton, Summit County, Ohio, - - - - 523

Cesarian operation. By John Travis, M. D., of Mansfield, Tenn., - 524

Friendly hints to a Reviewer.—Something more on moist hot air. By Samuel Jackson, M. D., (formerly of Northumberland.) - 526

### BIBLIOGRAPHICAL NOTICES.

The Principles and practice of Dental Surgery. By Chapin A. Harris, M. D., D. D. S., Professor of the Principles and Practice of Dental Surgery in the Baltimore College, &c. &c., - - 542

General Therapeutics and Materia Medica; adapted for a Medical Text Book. By Robley Dunglison, M. D., Professor of Institutes of Medicine in Jefferson Medical College, &c. &c., - 543

Medical Jurisprudence. By Alfred Taylor, F. R. S., Licentiate of the Royal College of Physicians, Member of the Royal College of Surgeons, Professor of Medical Jurisprudence and Chemistry in Guy's Hospital, - - - - - - 544

Report of the Trial. The People vs. Dr. Horatio N. Loomis, for libel. Tried at the Erie Court of Oyer and Terminer, June 24, 1850. 544

### EDITORIAL.

Sydenham Society of London, - - - - - 545

Deaths in Philadelphia from July 20th to August, 24th, 1850. Reported by Mr. James Aitken Meigs, Student of Medicine, - 547

### RECORD OF MEDICAL SCIENCE.

### ANATOMY AND PHYSIOLOGY.

Sensibility of the teeth explained on Hydrostatic principles. By J. Neill, M. D., Demonstrator of Anatomy in the University of Pennsylvania, - - - - - - 548

An Exposition of M. Bernard's Discovery of the Renal Circulation. By Juriah Harris, M. D., of Georgia, - - - - 549

### PATHOLOGY AND PRACTICE OF MEDICINE.

Of the peculiarities of Structure in Individual Parts and Organs and particular regions of the Body in the Tuberculous Predisposition. By Henry Ancele, Esq., - - - - 553

SURGERY.

Lithotomy in a very young Boy. Under the care of Mr. Avery, - 556

MATERIA MEDICA AND THERAPEUTICS.

Nutritive Properties of Coffee. - - - - - 558
On a probable danger from the use of Cod-Liver Oil. By Dr. Benson, 559
New Adhesive Dressing. By Dr. Mellez, - - - - 559
Various Surgical Uses of Vulcanized India-Rubber, - - 559
On the Use of Acetic Acid in Preventing the Spread of Scarlatina. By Dr. J. W. Webster, Consulting Physician to the St. George's and St. James's Dispensary, - - - - - 560

